# Differential brain responses to affective sounds in misophonia and hyperacusis: A task-based fMRI approach

**DOI:** 10.3758/s13415-026-01435-z

**Published:** 2026-04-14

**Authors:** Namitha Jain, Shagun Ajmera, Somayeh Shahsavarani, Rafay A. Khan, Gibbeum Kim, Howard Berenbaum, Fatima T. Husain

**Affiliations:** 1 Department of Speech and Hearing Science, University of Illinois Urbana-Champaign, 901 S Sixth St, Champaign, IL 61820 USA; 2 Beckman Institute for Advanced Science & Technology, University of Illinois Urbana-Champaign, Urbana, IL 61801 USA; 3 Neuroscience Program, University of Illinois Urbana-Champaign, Urbana, IL 61801 USA; 4 Department of Audiology, San Jose State University, San Jose, CA 95192 USA; 5 Department of Psychology, University of Illinois Urbana-Champaign, Champaign, IL 61820 USA

**Keywords:** Emotion, Functional connectivity, Neural network, Auditory, Sound sensitivity disorders, Misophonia

## Abstract

Humans are naturally attuned to emotionally salient sounds, such as screams signaling danger, which trigger survival-related responses. In sound-sensitivity disorders, such as misophonia and hyperacusis, everyday sounds provoke intense emotional and behavioral reactions. Misophonia is typically triggered by specific sounds, such as chewing, whereas hyperacusis involves hypersensitivity to sounds above certain intensity thresholds. Because these disorders share overlapping symptoms and frequently co-occur, disentangling their neural bases is essential for improving diagnosis and treatments. We recruited 91 young adults categorized into four groups: misophonia, hyperacusis, comorbid misophonia and hyperacusis, and controls. We conducted task-based fMRI, where participants listened to 90 emotionally valenced sounds from the International Affective Digitized Sounds-2 database and rated their valence during scanning. Whole-brain functional activation and seed-to-voxel functional connectivity analyses revealed both distinct and overlapping pattern of neural correlates associated with these disorders. The misophonia group, regardless of comorbid hyperacusis (relative to controls), showed hyperactivation in visual association areas and reduced connectivity between salience and visual networks during unpleasant versus neutral sound processing. This suggests atypical cross-modal sensory involvement. The hyperacusis group exhibited reduced connectivity between salience hubs and frontal control regions compared to misophonia and controls, indicating impaired top-down regulation. In contrast, this connectivity was preserved in misophonia for generally unpleasant sounds, suggesting intact regulation. The comorbid group showed neural patterns associated with both disorders. Overall, these findings reveal overlapping and disorder-specific neural patterns across sound tolerance profiles. Future research should combine neural and behavioral data to refine mechanistic models and guide targeted interventions.

**Supplementary Information:**

The online version contains supplementary material available at 10.3758/s13415-026-01435-z.

## Spanish

Los seres humanos están naturalmente atentos a sonidos con alta relevancia emocional, como los gritos que señalan peligro, los cuales desencadenan respuestas relacionadas con la supervivencia. En los trastornos de sensibilidad al sonido, como la misofonía y la hiperacusia, los sonidos cotidianos pueden provocar reacciones emocionales y conductuales muy intensas. La misofonía suele desencadenarse por sonidos específicos, como la masticación, mientras que la hiperacusia implica una hipersensibilidad a sonidos que superan cierto nivel de intensidad. Debido a que estos trastornos presentan síntomas superpuestos y con frecuencia coexisten, distinguir sus bases neurales es fundamental para mejorar el diagnóstico y el tratamiento. Se reclutó a 91 adultos jóvenes clasificados en cuatro grupos: misofonía, hiperacusia, ambos trastornos de forma comórbida y un grupo control. Se realizó una resonancia magnética funcional basada en tareas (fMRI), durante la cual los participantes escucharon 90 sonidos con valencia emocional de la base de datos *International Affective Digitized Sounds-2* y calificaron su valencia mientras eran escaneados. Los análisis cerebrales mostraron tanto patrones neuronales específicos como compartidos entre los trastornos. El grupo con misofonía, independientemente de la presencia de hiperacusia comórbida y en comparación con el grupo control, mostró hiperactivación en áreas de asociación visual y una conectividad reducida entre las redes de saliencia y las redes visuales durante el procesamiento de sonidos desagradables frente a sonidos neutros. Esto sugiere una participación sensorial intermodal atípica. El grupo con hiperacusia presentó menor conectividad entre las regiones de atención a estímulos relevantes y las zonas de control frontal, indicando dificultades en la regulación consciente de la percepción sonora. En misofonía, esta conectividad se mantenía para sonidos desagradables, lo que sugiere que la regulación está preservada. El grupo comórbido mostró patrones neurales asociados con ambos trastornos. En conjunto, estos resultados muestran que hay tanto patrones neuronales comunes como específicos de cada trastorno en la tolerancia al sonido. Futuras investigaciones deberían combinar información del cerebro y del comportamiento para mejorar los modelos que explican estos trastornos y desarrollar tratamientos más efectivos.

## Hindi

मनुष्य स्वाभाविक रूप से उन आवाज़ों के प्रति संवेदनशील होते हैं जिनमें भावनात्मक महत्व होता है, जैसे खतरे की चेतावनी देने वाली चिल्लाहट। ऐसी आवाज़ें हमारे शरीर की बचाव से जुड़ी प्रतिक्रियाओं को सक्रिय करती हैं। कुछ लोगों में ध्वनि संवेदनशीलता की समस्याएँ होती हैं, जैसे मिसोफोनिया और हाइपराक्यूसिस। इन लोगों के लिए सामान्य रोजमर्रा की आवाज़ें भी बहुत तीव्र भावनात्मक और व्यवहारिक प्रतिक्रिया पैदा कर सकती हैं। मिसोफोनिया अक्सर किसी खास आवाज़, जैसे खाने-पीने की आवाज़, से शुरू होती है, जबकि हाइपराक्यूसिस में तेज़ या तीव्र आवाज़ों के प्रति संवेदनशीलता बढ़ जाती है। ये दोनों समस्याएँ कभी-कभी एक साथ भी देखी जाती हैं। इसलिए इनके मस्तिष्क में होने वाले बदलावों को समझना इलाज और निदान के लिए जरूरी है। इस अध्ययन में हमने 91 युवा वयस्कों को चार समूहों में बाँटा: मिसोफोनिया वाले, हाइपराक्यूसिस वाले, दोनों समस्याओं वाले (सहविकारी), सामान्य लोग (नियंत्रण समूह). प्रतिभागियों ने इंटरनेशनल अफेक्टिव डिजिटाइज्ड साउंड्स-2 (IADS-2) डेटाबेस से 90 अलग-अलग भावनात्मक आवाज़ें सुनीं और स्कैनिंग के दौरान उन्होंने बताया कि उन्हें ये आवाज़ें कैसी लगीं। अनुसंधान में देखा गया कि जब मिसोफोनिया वाले लोग अप्रिय आवाज़ें सुनते हैं, तो उनके दिमाग के देखने वाले हिस्से ज्यादा सक्रिय हो जाते हैं । साथ ही, महत्त्व-निर्धारण तंत्र (salience network) और दृश्य नेटवर्क (visual network) के बीच तालमेल कम हो जाता है। इसका मतलब है कि उनका दिमाग सुनने और देखने की जानकारी को सामान्य से अलग तरीके से जोड़ता है। हाइपराक्यूसिस वाले लोगों में महत्त्व-निर्धारण तंत्र और मस्तिष्क के मुख्य कार्यकारी तंत्र (जो सोचने, ध्यान रखने और निर्णय लेने जैसी उच्चस्तरीय संज्ञानात्मक प्रक्रियाओं को संभालता है) के बीच कनेक्शन कम पाया गया। इसका मतलब है कि उनके दिमाग का उच्चस्तरीय नियंत्रण कमजोर है। इसके विपरीत, मिसोफोनिया वाले लोगों में यह कनेक्शन सामान्य बना रहता है, यानी उनका दिमाग नियंत्रण बनाए रखता है। जिन लोगों में दोनों समस्याएँ एक साथ होती हैं, उनके दिमाग में दोनों तरह के पैटर्न दिखाई देते हैं।” इन निष्कर्षों से पता चलता है कि कुछ मस्तिष्क पैटर्न केवल मिसोफोनिया या हाइपराक्यूसिस से जुड़े होते हैं, जबकि कुछ पैटर्न दोनों में सामान्य होते हैं। भविष्य में, मस्तिष्क और व्यवहार से जुड़े डेटा को एक साथ मिलाकर हम इन समस्याओं के बेहतर मॉडल और लक्षित इलाज विकसित कर सकते हैं।

## Introduction

Humans have evolved heightened sensitivity to certain sounds, such as screams or alarms, which act as survival cues by rapidly capturing attention and eliciting defensive responses (Arnal et al., 2015; Belin & Zatorre, 2015). In some individuals, this sensitivity extends maladaptively to everyday sounds, manifesting as decreased sound tolerance disorders. These include clinically distinct conditions, most notably hyperacusis and misophonia (Henry et al., 2022). Misophonia is characterized by intense negative emotional reactions to “trigger sounds,” often orofacial (e.g., chewing, sniffing) or repetitive sounds (e.g., pen clicking), driven primarily by context rather than loudness (Hansen et al., 2021; Swedo et al., 2022). Hyperacusis, by contrast, involves sensitivity to any sound that exceeds a certain loudness threshold, regardless of type, provoking discomfort or pain (Baguley, 2003; Henry et al., 2022). Both conditions impair quality of life, lead to avoidance behaviors, and frequently co-occur (Baguley, 2003; Brennan et al., 2024; Wu et al., 2014), highlighting the importance of delineating their neural correlates to inform targeted interventions.

Neuroimaging studies suggest that misophonia is associated with alterations in salience, emotion, and attention networks (Eijsker et al., 2021; Kumar et al., 2017; Schröder et al., 2019). The anterior insula, a key hub of the salience network, exhibits hyperactivity to trigger sounds, which increases with symptom severity (Kumar et al., 2017). It also exhibits increased coupling with emotion-processing (amygdala, hippocampus, ventromedial prefrontal cortex) and self-referential areas (posteromedial cortex) (Kumar et al., 2017). Another audio-visual stimulus-based study found hyperactivity in the anterior cingulate cortex and superior temporal gyrus (Schröder et al., 2019). A resting-state fMRI study further revealed altered intrinsic connectivity within ventral attention networks (Eijsker et al., 2021). Together, these findings indicate both exaggerated sound-evoked responses and trait-like, network-level changes correlated with misophonia presence.

Among the misophonic triggers, orofacial sounds are the most common, leading to the “hyper-mirroring” hypothesis which attributes misophonic responses to exaggerated mirroring of the actions producing them (Kumar et al., 2021). Supporting this, Kumar et al. (2021) reported increased resting-state connectivity of the ventral premotor cortex, a putative orofacial mirror-neuron region, with the planum temporale and anterior insula. In that same study, hyperactivation in the orofacial motor area during trigger-sound processing was also observed (Kumar et al., 2021). However, subsequent work by Hansen et al. (2022) found that misophonia engages broader sensory-motor cortices, rather than the selective involvement of orofacial motor areas, thus challenging hyper-mirroring hypothesis. This broader pattern suggests that misophonia may reflect a distributed network-level dysfunction involving sensory, motor, and salience systems, rather than a narrowly defined mirror-neuron mechanism or a purely auditory process.

As against the above, hyperacusis appears to be associated with heightened sound-evoked activity throughout the auditory pathway, including the superior olivary complex, inferior colliculus, medial geniculate body, and primary and secondary auditory cortices (Hofmeier et al., 2021; Koops & van Dijk, 2021). Such hyperresponsivity aligns with the central gain theory, which posits that reduced or altered peripheral input prompts the brain to upregulate neural gain, thereby producing exaggerated sound responses (Gu et al., 2010). Altered activation has also been observed in regions beyond the auditory cortices. For example, hyperactivation in the supplementary motor area during pure tone stimulation, likely reflects heightened motor readiness or preparatory responses to aversive or salient sounds (Makani et al., 2023). Furthermore, functional connectivity studies reveal enhanced coupling between auditory and limbic regions (hippocampus and amygdala) (Chen et al., 2015; Hofmeier et al., 2021), potentially indicating secondary salience-related responses to increased auditory gain. Taken together, these findings suggest that hyperacusis may be primarily associated with dysfunction within the auditory pathway, with non-auditory region engagement likely reflecting secondary effects of heightened auditory processing.

Despite these insights, existing neuroimaging evidence distinguishing misophonia from hyperacusis is largely indirect, having been derived from separate studies that differ substantially in participant selection, stimulus characteristics, and analytic approaches. Most prior work has examined either misophonia (Eijsker et al., 2021; Hansen et al., 2022; Kumar et al., 2017; 2021; Schröder et al., 2019) or hyperacusis (Chen et al., 2015; Hofmeier et al., 2021; Koops & van Dijk, 2021; Makani et al., 2023) in isolation, without accounting for comorbidity (Brennan et al., 2024) or applying clear exclusion criteria, thereby risking conflation of neural associations. Furthermore, misophonia studies often use nonstandardized, researcher-biased orofacial sounds (Kumar et al., 2017, 2021; Schröder et al., 2019), whereas hyperacusis studies typically employ pure tones (Koops & van Dijk, 2021), creating divergent stimulus sets that limit cross-study comparison and obscure disorder-specific versus shared mechanisms.

To address above-described gaps, the present study employed standardized, emotionally valenced sounds and a design allowing direct comparison of functional activity and connectivity across misophonia, hyperacusis, and comorbid groups. Unlike prior work focused solely on misophonia trigger sounds or pure tones in hyperacusis, our design broadens the scope to examine general affective sound processing in both conditions. This broader approach enables us to determine whether neural differences observed in misophonia extend beyond trigger-specific reactions and to explore affective-related, nonauditory pathways associated with hyperacusis. We also conducted a narrow analysis focused on regions of interest identified in previous research—such as the anterior insula, linked to misophonia triggers (Kumar et al., 2017, 2021), and auditory cortices, associated with sound-based hyperacusic responses (Hofmeier et al., 2021). Although misophonic triggers were not presented, the anterior insula was included as a region of interest given its broader role in salience detection and affective evaluation of aversive stimuli. By examining activation in these regions and their functional coupling during processing of nontrigger, generally unpleasant sounds, we aimed to clarify shared and distinct neural correlates associated with misophonia and hyperacusis.

## Materials and methods

### Participants

Participants for the current study were recruited through a multi-stage process. We first conducted a large-scale online survey among undergraduate and graduate students between the ages of 18 and 25 at the University of Illinois Urbana-Champaign. The survey included the Khalfa Hyperacusis Questionnaire (HQ: Khalfa et al., 2002), the Misophonia Questionnaire (MQ: Wu et al., 2014), as well as the tinnitus and hearing health survey. More details of the survey design and procedures are described in Brennan et al. (2024). Participants whose questionnaire scores suggested possible hyperacusis and/or misophonia were invited for in-person psychological and audiological assessments.

Misophonia was diagnosed using a structured clinical interview based on the criteria proposed by Lewin et al., (2015). Their criteria for misophonia classification included sound-specific hypersensitivity, associated emotional and physiological responses, avoidance behaviors, and functional impairment not explained by other psychiatric conditions. Although newer consensus criteria have since been proposed (Swedo et al., 2022), data collection for our study had already begun. These updated criteria largely build on the same core features of Lewin et al. (2015) but offer greater descriptive detail. The structured interviews for misophonia and screening for other affective disorders, such as major depressive disorder (MDD) and obsessive compulsive disorder (OCD), were administered by a doctoral candidate in clinical psychology under the supervision of a licensed clinical psychologist. Hyperacusis was defined as a hyperacusis questionnaire score greater than 22 (Aazh & Moore, 2017).

A total of 92 participants were recruited for the task-based fMRI portion of the study and classified into four groups: misophonia only (MISO; *n* = 29), hyperacusis only (HY; *n* = 14), comorbid misophonia and hyperacusis (MiHy; *n* = 24), and healthy controls (CTR; *n* = 25) with neither disorder. Age and sex distributions were examined across groups and did not differ significantly (see *Results*). Demographic characteristics are presented in Table 1. The study was approved by the University of Illinois Urbana-Champaign Institutional Review Board (IRB24-1118).

**Table 1:** Demographic and clinical characteristics of participants across groups

	Misophonia (MISO)	Hyperacusis (HY)	Comorbid (MiHy)	Control (CTR)
N	29	14	24	25
Sex	20 females	9 females	18 females	13 females
Age	20.86 (2.07)	19.79 (1.76)	20.17 (1.79)	20.2 (2.04)
Misophonia Diagnosis	Yes	No	Yes	No
HQ scores*	14.04 (5.33)	26.07 (2.95)	28.92 (5.01)	8.8 (4.46)
MQ scores*	58.79 (15.44)	65.64 (9.72)	74.75 (10.33)	39 (11.08)
MQ severity*	7.46 (3.8)	8.64 (2.24)	10.38 (2.12)	4.88 (4.42)
PTA	7.61 (6.15)	7.01 (2.16)	7.01 (2.76)	8.32 (2.91)
LDL*	90.04 (22.46)	83.51 (7.14)	82.95 (10.18)	92.13 (9.00)
MDD ratings (absent (0), subthreshold (1), at or above threshold (2))	9 (0), 19 (1), 0 (2), 1 (unknown)	6 (0), 8 (1), 0 (2)	7 (0), 12 (1), 0 (2), 5 (unknown)	15 (0), 10 (1), 0 (2)
OCD ratings (no (0), yes (1))	20 (0), 7 (1), 2 (unknown)	13 (0), 1 (1)	13 (0), 9 (1)	23 (0), 2 (1)

*Notes.* Values are presented as mean (SD) unless otherwise indicated. *Factors that were significantly different across groups. Misophonia diagnosis: determined through a structured clinical interview; HQ scores: hyperacusis questionnaire scores; MQ = misophonia questionnaire; PTA = pure-tone average (dB HL) across 0.5, 1, 2, and 4 kHz; LDL = loudness discomfort level (dB HL), averaged across 0.5 kHz, 4 kHz, and speech stimuli; MDD = Clinician’s ratings for major depressive disorder based on Structural Clinical Interview for DSM-5. OCD ratings: Obsessive Compulsive Disorder ratings based Structural Clinical Interview for DSM-5

As neuroimaging research has advanced, standards for estimating adequate sample sizes have become more established (Desmond & Glover, 2002; Mumford, 2012). Accordingly, the present study aimed to include a minimum of 20 participants per group to provide reasonable statistical power for detecting group-level effects. Sample sizes of this magnitude are comparable to those used in prior task-based fMRI studies of misophonia (Kumar et al., 2017, 2021; Schröder et al., 2019) and hyperacusis (Hofmeier et al., 2021), which have reported robust group differences. The recruitment target of 20 per group was achieved for all groups except the HY group. Recruitment of a pure hyperacusis sample was constrained by the relatively low prevalence of hyperacusis in young adults with normal hearing (~5%), compared with higher prevalence estimates in middle-aged and older adults (15–20%) (Ren et al., 2021; Smit et al., 2021; Yilmaz et al., 2017). Furthermore, hyperacusis often co-occurs with misophonia (Brennan et al., 2024), which was reflected in our sample where many individuals meeting hyperacusis criteria also met misophonia criteria and were thus classified as having both conditions.

### Audiometric evaluation

All participants completed a comprehensive audiological assessment in a sound-attenuating booth. Pure-tone thresholds were measured at standard frequencies (0.25–8 kHz) and extended high frequencies (9–16 kHz). Pure tone averages (PTA) were calculated across 0.5, 1, 2, and 4 kHz. All participants demonstrated normal hearing sensitivity (PTA ≤ 25 dB HL), except for two individuals in the MISO group whose averages (25.83 and 26.67 dB HL) fell within the mild hearing loss range. These participants were retained, as all task-based fMRI auditory stimuli were presented at individually adjusted maximum comfortable volume to ensure audibility.

During audiometric testing, loudness discomfort levels were recorded at 0.5 kHz, 4 kHz, and using spondee speech stimuli. Middle-ear status was evaluated using tympanometry; only participants with type A tympanograms were included. Acoustic reflexes were also assessed, although some participants could not complete this portion of testing due to sound sensitivity.

### fMRI data acquisition

Functional and structural MRI data were collected using a 3 T Siemens Prisma scanner with a 20-channel head coil at the Biomedical Imaging Center, Beckman Institute, University of Illinois Urbana-Champaign. High-resolution anatomical images were acquired using a T1-weighted MPRAGE sequence (TR = 2,300 ms, TE = 2.32 ms, flip angle = 8°, voxel size = 0.9 × 0.9 × 0.9 mm, slice thickness = 0.9 mm, 192 sagittal slices). Functional images were obtained using a continuous acquisition paradigm (TR = 1,500 ms, TE = 30 ms, flip angle = 73°, slice thickness = 3 mm, voxel size = 3 × 3 × 3 mm, 40 axial slices (no gap), field of view = 210 mm). In total, 459 volumes were collected during a single run.

### Experiment

#### Stimuli

Auditory stimuli were selected from the International Affective Digitized Sounds-2 (IADS-2) database, which contains 167 sounds rated for valence (1 = very unpleasant, 9 = very pleasant) and arousal (1 = not at all arousing, 9 = very arousing) (Bradley & Lang, 2007). We selected 89 sounds, comprising 30 unpleasant, 30 pleasant, and 29 neutral stimuli (see Supplementary 1). Three pseudorandomized lists were created, each consisting of 90 stimuli, with one pleasant sound presented twice per list. Each participant was presented with one list at random. Normative ratings for the selected sounds were as follows: pleasant (valence: 6.85 ± 1.36, arousal: 6.49 ± 1.31), unpleasant (valence: 2.78 ± 0.58, arousal: 6.96 ± 0.32), and neutral (valence: 4.85 ± 0.59, arousal: 4.86 ± 0.57). Valence and arousal scores significantly differed across all categories. These stimuli have been previously used in our lab in fMRI studies of tinnitus (Carpenter-Thompson et al., 2014, 2015a, 2015b), demonstrating their suitability for research on clinical auditory populations. Standardized affective sounds were selected that did not include commonly reported misophonic trigger sounds, such as primarily orofacial noises. Importantly, using standardized affective sounds avoids reliance on idiosyncratic triggers, ensures consistent emotional engagement across participants, and allows controlled comparisons between groups.

#### Design

Participants first completed practice trials with IADS-2 sounds not used in the experiment, to familiarize them with the task. During scanning, they listened to 90 sounds each 6 s, presented at their individually adjusted maximum comfortable volume. Participants were instructed to rate each sound as pleasant (P), neutral (N), or unpleasant (U) using a button press with their dominant hand as soon as they felt confident. Responses and reaction times were recorded. Participants were allowed to revise their responses during the 6-s window, with only the last recorded rating used for analysis. Each trial was separated by a 1.5-s intertrial interval consistent with prior affective sound-processing paradigms in misophonia that used a 2-s intertrial interval (Schröder et al., 2019). Physiological data were recorded concurrently during the experiment. Heart rate was measured via photoplethysmography from the index finger of the nondominant hand, and respiration was monitored using a pneumatic chest belt.

Stimuli and instructions were presented using Presentation 23.1 software (www.neurobs.com). Sounds were delivered via MR-compatible Avotec headphones (Avotec Inc., Stuart, FL). To reduce scanner noise, participants wore both earplugs and MR-safe noise-canceling headphones. Participants were informed that they could terminate the session at any time if they experienced discomfort.

### Data preprocessing

Functional MRI data were preprocessed using SPM12 (Wellcome Centre for Human Neuroimaging, London, UK; www.fil.ion.ucl.ac.uk/spm/software/spm12/), and MATLAB (MathWorks, version 2024a). Preprocessing steps included slice-timing correction referenced to the second slice acquired in an interleaved order, realignment for head motion correction, and co-registration of the mean functional image to the high-resolution T1-weighted anatomical image. The co-registered T1-weighted images were normalized to the Montreal Neurological Institute (MNI) template space using a non-linear warp transformation, and the resulting normalization parameters were applied to the functional data. The normalized fMRI images were resampled to 2-mm isotropic voxels and smoothed using an 8×8×8 mm^3^ full-width at half-maximum Gaussian kernel.

Raw physiological recordings, including respiratory and cardiac signals, were processed using the PhysIO toolbox (www.nitrc.org/projects/physio/). The preprocessing output included nine respiratory and seven cardiac parameters, which were subsequently used as nuisance regressors in brain activation analysis.

Functional connectivity analyses were conducted in the CONN toolbox (version 22.v2407; web.conn-toolbox.org). Preprocessed anatomical and functional images were entered into the CONN pipeline. Outlier volumes were identified using artifact detection tools, with scrubbing applied to volumes showing framewise displacement above 0.9 mm or global BOLD signal changes exceeding 5 standard deviations. One participant was excluded for excessive motion, as approximately 27% (124) of volumes were removed during scrubbing. Across the remaining participants, an average of 6 (1.3%) volumes were scrubbed, ranging from 0 to 63 (0–13.7%) volumes per individual. Denoising included regression of six head motion parameters and their first-order derivatives, seven white matter and five cerebrospinal fluid principal components (aCompCor), and ART-identified outlier volumes. aCompCor effectively captures physiological cardiac and respiratory fluctuations, providing estimates comparable to external recordings such as RETROICOR (Behzadi et al., 2007; Caballero-Gaudes & Reynolds, 2016). Global signal regression was not applied, as it can introduce spurious negative correlations, and potentially obscure group differences and brain–behavior relationships (Gotts et al., 2013; Murphy et al., 2009). These steps minimized motion- and physiology-related confounds and reduced positive bias in functional connectivity estimates. Finally, linear drifts were removed by band-pass filtering (0.008–0.1 Hz).

Seed regions of interest (ROI) were created using MarsBaR (Brett et al., 2002; version 0.45) as spherical ROIs with an 8 mm radius, centered on the MNI coordinates listed in Table 2. This radius was chosen to balance anatomical specificity and signal reliability, capturing a sufficient portion of the target region while minimizing inclusion of neighboring voxels (Eickhoff et al., 2006). Table 2 also details the networks and corresponding seed locations. ROIs for the auditory (left superior temporal gyrus and right planum temporale), salience (bilateral anterior insula and right anterior cingulate cortex [ACC]), and motor networks were selected based on prior misophonia and hyperacusis studies (Kumar et al., 2017, 2021; Makani et al., 2023; Schröder et al., 2019). The left ACC seed was taken from (Zhou et al., 2016) to allow bilateral representation. Coordinates for the remaining auditory network seeds (bilateral primary auditory cortices), as well as ROIs in the default mode network, frontoparietal network, and bilateral amygdalae, were drawn from previous tinnitus research conducted in our lab (Schmidt et al., 2013; Shahsavarani et al., 2021).

**Table 2:** Regions-of-interest used for functional activation and connectivity analyses, organized by network, with corresponding MNI coordinates

Network	ROI	Hemisphere	MNI coordinates		
			x	y	z
***Auditory***	Primary Auditory cortex	R	55	−22	9
	Primary Auditory cortex	L	−41	−27	6
	Superior Temporal gyrus	L	−60	6	−4
	Planum temporale	R	30	−34	20
***Salience***	Anterior Insula	R	39	29	−3
	Anterior Insula	L	−41	6	0
	Anterior Cingulate	R	4	44	16
	Anterior Cingulate	L	−5	47	11
***Default Mode***	Ventromedial prefrontal cortex	L	−3	44	−2
	Ventromedial prefrontal cortex	R	8	59	19
	Precuneus	L	−6	−54	42
***Central Executive***	Dorsolateral prefrontal cortex	R	41	38	30
	Dorsolateral prefrontal cortex	L	−43	33	28
***Emotion processing***	Amygdala	R	18	−7	−17
	Amygdala	L	−17	−2	−24
***Sensorimotor***	Supplementary Motor Area	R	8	−10	60
	Ventral premotor cortex	R	60	12	24
	Primary motor/sensory	L	−18	−16	78

R = right; L = left

### Data analysis

#### Behavioral

Behavioral data were analyzed using IBM SPSS Statistics (Version 22). Normality tests indicated that the data were not normally distributed across all groups (*p* < 0.05). Group differences in age, hearing thresholds, HQ, MQ total scores, MQ severity scale, and loudness discomfort levels were assessed using the nonparametric Kruskal-Wallis test. Group differences in sex, MDD ratings, and OCD ratings were evaluated using a chi-square test of independence. Behavioral valence ratings and reaction times were analyzed using linear mixed-effects models, which accommodate unbalanced data and deviations from normality (Schielzeth et al., 2020). For valence ratings, the model included Group (MISO, HY, MiHy, CTR) as a between-subjects factor and Valence Category (Unpleasant, Neutral, Pleasant) as a within-subjects factor. Reaction times were analyzed in a separate LME with the same factor structure. Bonferroni correction was applied for post hoc comparisons. Statistical significance was set at *p* < 0.05. To examine how participants reclassified predefined valence categories, we conducted crosstabulations comparing participant-rated valence categories to the original sound categories. For each predefined category, contingency tables summarized the frequency of reclassification by group. Group differences in these patterns were assessed using chi-square tests of independence.

#### Functional activation

For first-level modeling, trials were classified based on each participant’s subjective valence ratings of the sounds rather than the normative IADS-2 categories. Trials were labeled as P, N, and U according to individual responses. Missing responses were rare; most participants missed only one to three trials, with just two participants missing 10 and 18 trials, yielding an overall missing rate of ~1.5%. Missing trials were imputed using the group-level modal response for each stimulus. First-level fixed effects analyses across the whole brain were performed on each subject’s smoothed images to generate P > N and U > N contrast images. The design matrix included six head motion parameters and sixteen physiological regressors (respiratory and cardiac) as covariates of no interest. Because Pleasant-rated trials were relatively rare (~11% on average), the P > N contrast was excluded from group-level whole-brain analyses to avoid spurious findings.

Second-level analysis was conducted only for the U > N contrast. Group differences were modeled within an ANOVA framework with group (MISO, HY, MiHy, CTR) as a between-subjects factor, allowing for independence and unequal variance assumptions. Given the relatively small sample size in the HY group (N = 14), statistical inference was performed using nonparametric permutation testing in SnPM13 (nisox.org/Software/SnPM13/) with 5,000 permutations. A cluster-defining threshold of *F* = 5.93 (*p* = 0.001, uncorrected; df = 3, 87) was applied, and cluster-level family-wise error (FWE) correction was used at *p* < 0.05. Planned follow-up two-sample t-tests also employed 5,000 permutations, with a cluster-defining threshold of *t* = 3.26 (*p* = 0.001, uncorrected) and cluster-level FWE correction at *p* < 0.0084 (df = [1, 51]).

Complementing the whole-brain analyses, ROI activation was examined using MANOVA for each functional network (e.g., auditory, salience, somatosensory, default mode). All ROIs within a given network served as dependent variables (see Table 2), with group as the independent factor. Because ROI analyses are more focused and hypothesis-driven, they offer greater sensitivity than whole-brain tests, thus, we included both U > N and P > N contrasts as within-subject factors. Pillai’s trace was used to assess multivariate significance due to violation of covariance homogeneity (significant Box’s M), providing a robust statistic (Field, 2024; Olson, 1974). Family-wise error correction at *p* < 0.05 was applied to control for multiple comparisons.

#### Functional connectivity

Seed-based functional connectivity analyses were conducted using the CONN toolbox for MATLAB. Following preprocessing and denoising (see Data Preprocessing section), first-level generalized psychophysiological interaction models were constructed using bivariate regression. Seeds were defined as 8-mm radius spheres centered on the coordinates listed in Table 2. For each seed, seed-to-voxel connectivity maps were generated for the U > N contrast.

At the second level, a multivariate F-test was performed for each functional network using nonparametric statistics with 5,000 permutations to assess overall group effects. Networks showing significant multivariate effects underwent follow-up univariate F-tests and pairwise group comparisons, also using 5,000 permutations. For networks with multiple ROIs, Bonferroni correction was applied to adjust the significance threshold at cluster-level (*p* < 0.05/number of ROIs). Post-hoc comparisons were additionally corrected for FWE.

Exploratory Spearman rank correlation analyses were performed to examine associations between misophonia symptom severity (MQ total scores and MQ severity scale) and task-related activation as well as seed-to-voxel functional connectivity measures. Multiple comparisons were corrected by using Bonferroni adjustment.

## Results

### Behavioral analysis

#### Demographics

Nonparametric Kruskal-Wallis tests and chi-square tests were used to assess group differences in age, PTA, loudness discomfort level, and sex distribution. Age (*H*(3, N = 92) = 4.85, *p* = 0.184), PTA (*H*(3, N = 92) = 4.03, *p* = 0.259), sex distribution (χ^2^(3, N = 92) = 3.61, *p* = 0.307), MDD ratings (χ^2^(3, N = 92) = 4.58, *p* = 0.205), and OCD ratings (χ^2^(3, N = 92) = 5.71, *p* = 0.321) did not differ significantly across groups. Loudness discomfort level (averaged across 1 kHz, 4 kHz, and speech stimuli) showed a significant group effect (*H*(3, N = 92) = 14.33, *p* = 0.002). Post-hoc tests with Bonferroni correction indicated that the HY group (adjusted *p* = 0.003) and MiHy group (adjusted *p* = 0.039) groups had significantly lower loudness discomfort levels than the CTR group, confirming their hyperacusis status and supporting the correspondence between hyperacusis questionnaire scores and loudness discomfort measures.

HQ scores also revealed robust group differences (H(3, N = 92) = 70.27, *p* < 0.001). CTR exhibited significantly lower HQ scores than MISO (adjusted *p* = 0.0087), HY (adjusted *p* = 0.0001), and MiHy (adjusted *p* = 0.0001). As expected, MISO scored lower than both HY and MiHy (adjusted *p* = 0.0001), while no difference was observed between the HY and MiHy groups (adjusted *p* = 0.644). Groups further differed on MQ total scores (H(3, N = 92) = 50.95, *p* < 0.001) and MQ severity scores (H(3, N = 92) = 28.61, *p* < 0.001). CTR reported significantly lower MQ total and severity scores than all clinical groups (all adjusted *p* ≤ 0.001). The MiHy group showed higher MQ total scores than MISO (adjusted *p* = 0.007). Interestingly, the HY did not differ from MISO (adjusted *p* = 0.454). A similar pattern emerged for MQ severity where the CTR had significantly lower scores compared to MISO (adjusted p = 0.049), HY (adjusted p = 0.0019), and MiHy (adjusted *p* = 0.0001). Further, MiHy had higher scores compared with MISO group (adjusted *p* = 0.035). The MISO group did not differ from HY group (adjusted *p* = 1.000).

#### Emotion task

##### Valence rating

**Fig. 1:**
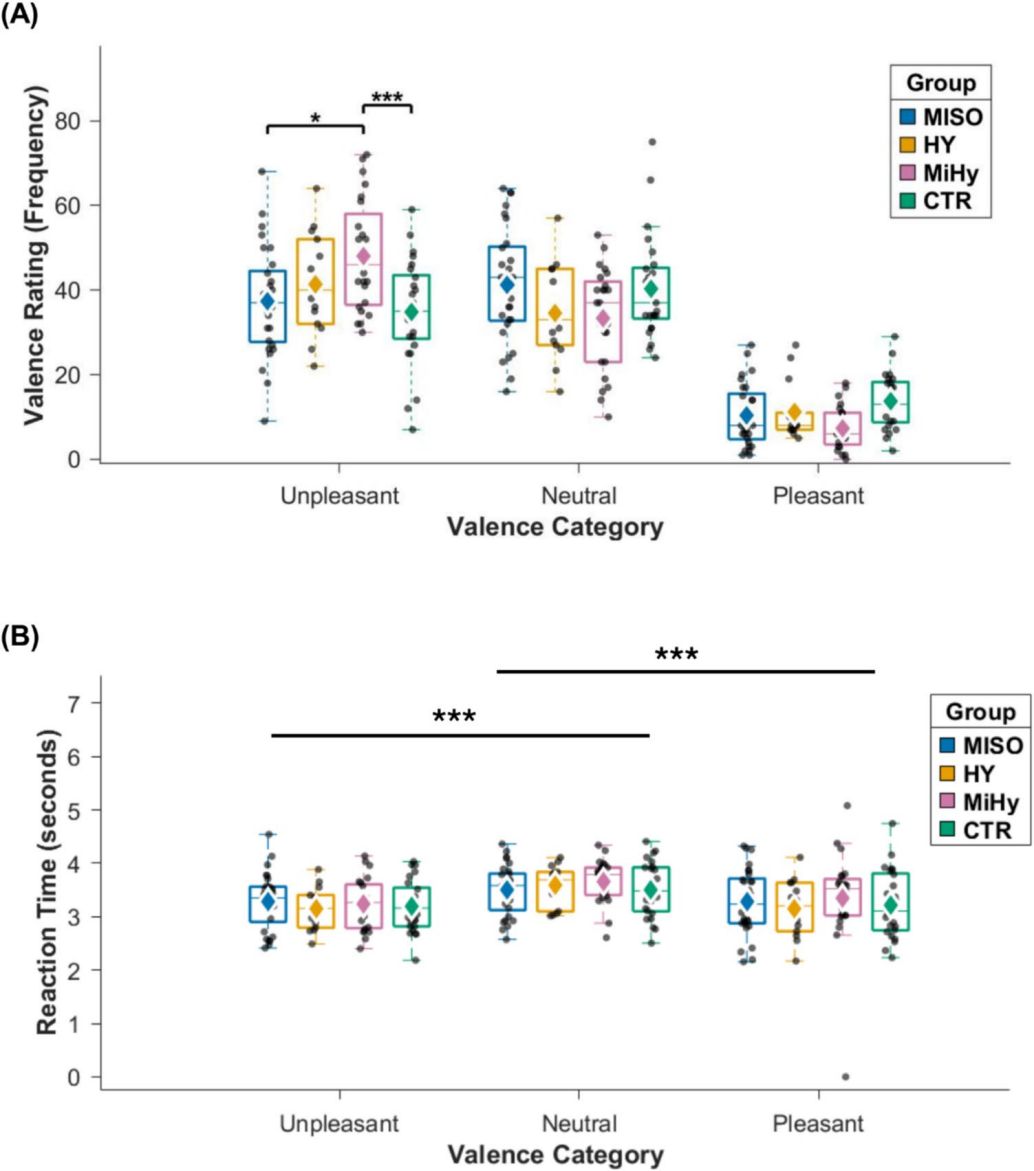
Boxplots of (**A**) valence ratings and (**B**) reaction times for unpleasant, neutral, and pleasant valence categories across participant groups: Misophonia (MISO), Hyperacusis (HY), Comorbid (MiHy), and Control (CTR). Valence ratings represent the frequency of ratings out of all sounds within each valence category. Reaction times represent mean response times across trials. The diamonds in the box plots indicate group means. *p < 0.05; ** *p*< 0.01; *** *p*< 0.001*.*

A linear mixed-effects model was used to assess group differences in the frequency of sounds rated across the three valence categories (U, N, P). Results revealed significant main effects of valence category (*F*(2,240) = 178.761, *p* < 0.0001), as well as a significant group × valence category interaction (*F*(6,240) = 5.862, *p* < 0.0001). Post-hoc tests for the interaction effect with Bonferroni correction showed that the MiHy group rated significantly more sounds as unpleasant than both the MISO (*p* = 0.0026) and CTR groups (*p* = 0.0002; Fig. 1A). No significant group differences were observed for neutral or pleasant sounds. The MiHy group’s higher unpleasant ratings may reflect heightened aversive perception. It is also important to note that, although we selected 30 sounds per valence category based on normative ratings, participants’ ratings across all groups were predominantly unpleasant.

Chi-square analyses revealed significant group differences in the reclassification of predefined unpleasant, neutral, and pleasant sounds based on subjective valence ratings (all *p* < 10⁻⁸). Standardized residuals from these analyses indicated that the MiHy group was significantly more likely than other groups to classify predefined neutral and pleasant sounds as unpleasant. Specifically, MiHy participants rated predefined pleasant sounds as unpleasant more often and as pleasant less often than expected. In contrast, CTR participants rated predefined pleasant sounds as pleasant more often than expected. For predefined neutral sounds, the MiHy group more often classified them as unpleasant and less often as neutral or pleasant. In contrast, CTR participants tended to keep neutral ratings and less frequently labeled these sounds as unpleasant. Overall, these results indicate that the MiHy group shows a consistent negative bias when evaluating predefined neutral and pleasant stimuli.

##### Reaction time

A linear mixed-effects model revealed no significant main effect of group or group × valence interaction. Only a significant main effect of valence category was observed (*F*(2,176) = 15.891, *p* < 0.0001), and post hoc comparisons with Bonferroni correction showed that all groups responded faster to affective sounds (unpleasant and pleasant) than to neutral sounds (*p* < 0.0001; Fig. 1B). It is important to note that participants were allowed to revise their responses, but only their final rating was used for analysis. On average, each participant changed their rating at least once for four to five stimuli. These revisions in responses may have contributed to the lack of group differences in reaction times.

### Functional activation analysis

#### Whole-brain analysis

**Fig. 2:**
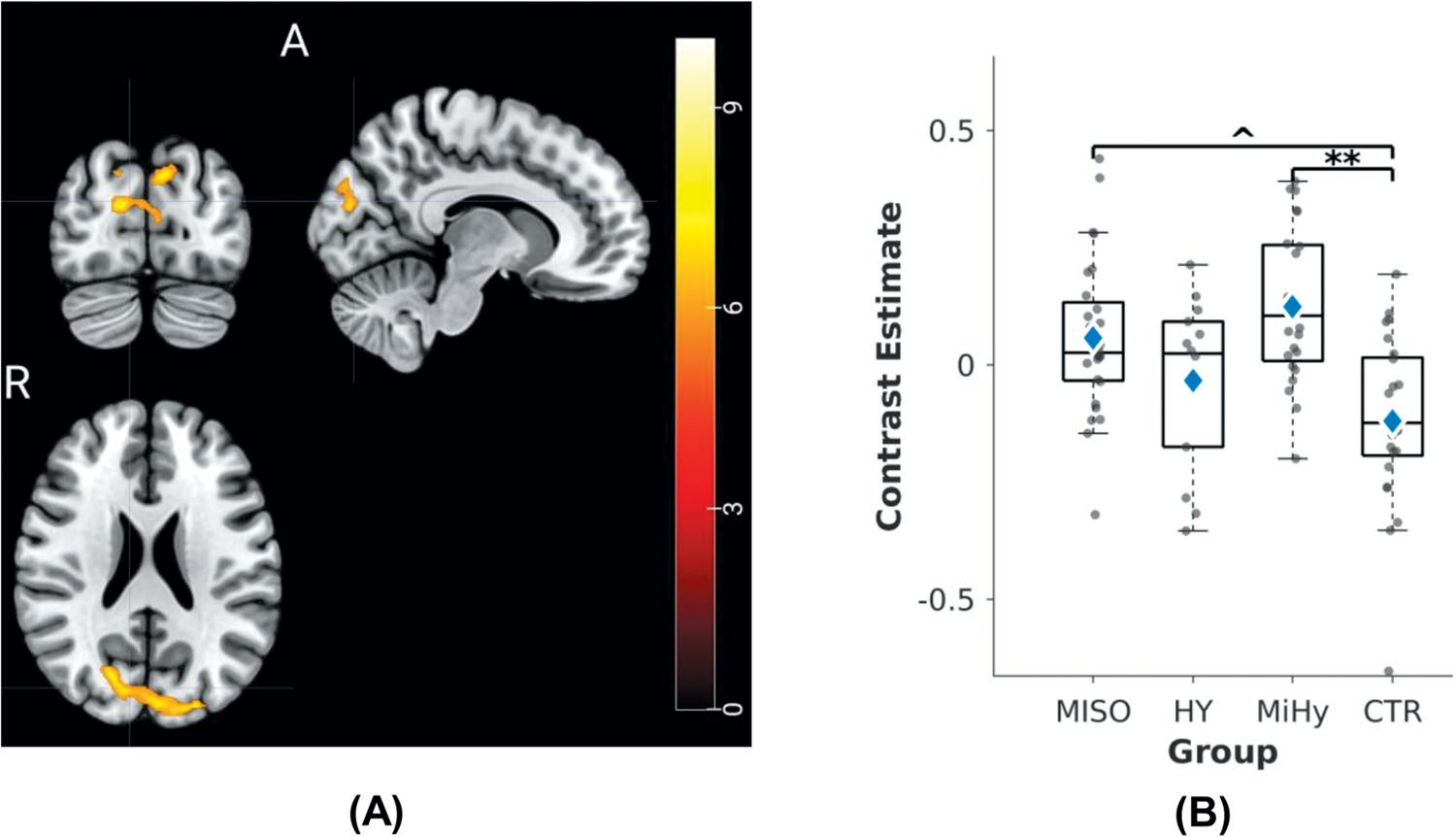
Whole-brain ANOVA results (with 5000 permutations) for the contrast Unpleasant > Neutral across four groups: misophonia (MISO), hyperacusis (HY), comorbid (MiHy), and control (CTR) (*N* = 91). (**A**) Significant cluster observed in the left visual association cortex (peak MNI: −20, −90, 28), extending bilaterally into secondary visual areas (cluster-level FWE-corrected *p* < 0.05; k = 962 voxels). Statistical maps are shown in coronal, sagittal, and transverse views, with A and R indicating anterior and right orientations. (**B**) Box plots of contrast estimates at the peak voxel across groups. Blue diamonds indicate group means. Statistical significance is reported after Bonferroni correction for multiple pairwise comparisons. *p < 0.05; **p < 0.01; ***p < 0.001; ^p denotes pairwise comparisons that were significant before Bonferroni correction.

A nonparametric ANOVA framework with 5,000 permutations on the U>N contrast identified a significant cluster surviving cluster-level FWE correction (*F*(3,87) = 11.39; p_FWE_ = 0.0038; *k* = 962) at peak MNI coordinates −20, −90, 28 (left visual association cortex). The cluster extended bilaterally into secondary visual areas (Fig. 2A). Pairwise comparisons revealed that the MISO group exhibited greater activation relative to CTR group in the cerebellum (*T* = 5.28; p_FWE_ = 0.0028; *k* = 542 voxels; peak MNI coordinates = −10, −72, −16). The MiHy group exhibited increased activation relative to CTR in the left visual association area (*T* = 5.47; p_FWE_ = 0.0006; *k* = 452; peak MNI coordinates = −22, −90, 26), extending bilaterally into secondary visual regions (Fig. 2B). Additionally, the MISO also showed increased activation in the left secondary and associative visual cortices (*T* = 4.35; p_FWE_ = 0.0216; *k* = 427; peak MNI coordinates: −24, −88, 28) relative to CTR but did not survive Bonferroni correction and are therefore reported as trends. The HY group showed no significant differences compared to other groups. These results are summarized in Table 3.

**Table 3:** Whole-brain and region-of-interest (ROI) activation results for the Unpleasant > Neutral contrast across four groups (misophonia only [MISO]; hyperacusis only [HY]; comorbid misophonia and hyperacusis [MiHy]; controls [CTR])

Group Comparison	Region	Hemisphere	MNI			Cluster Size (k)	*T*
			x	y	z		
MISO > CTR	Cerebellum	Left	−10	−72	−16	542	5.28
	Visual Association Area	Left	−24	−88	28	427	4.35*
MiHy > CTR	Visual Association Area	Left	−22	−90	26	452	5.47
MISO > CTR	[ROI] Ventromedial Prefrontal Cortex	Left	−			−	2.01*

*Notes.* Threshold: voxel-level *p* < 0.001 and cluster-level FWE-corrected *p* < 0.05 for whole-brain analyses. ROI: region-of-interest analyses; no MNI coordinates or cluster sizes are reported because these analyses are based on mean contrast estimates within predefined anatomical masks. *Results significant before pairwise Bonferroni correction for multiple *t*-tests

#### Region of interest analysis

**Fig. 3:**
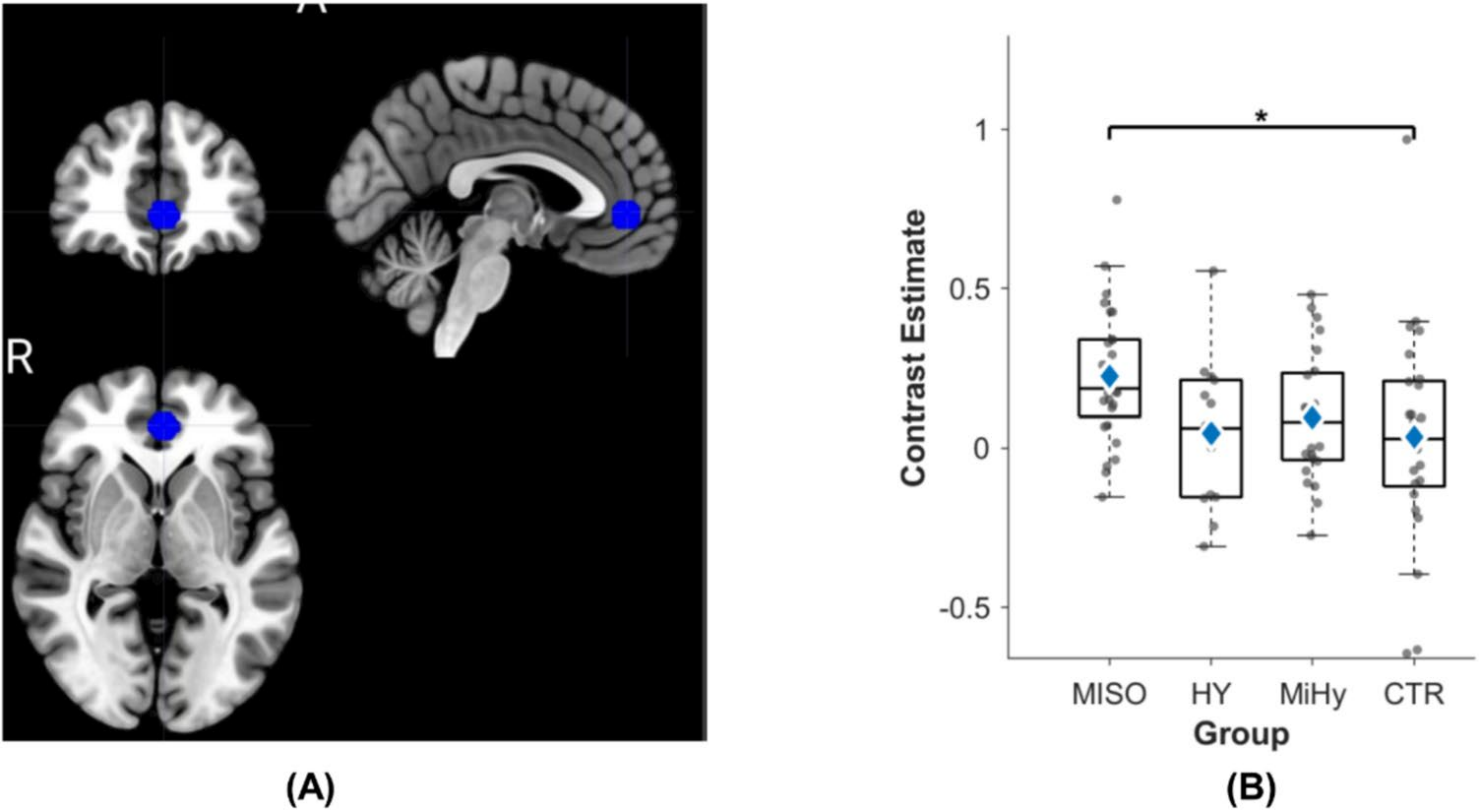
(**A**) Left ventromedial prefrontal cortex (vmPFC) seed region (blue) used for region-of-interest analysis of default mode network (DMN) regions. (**B**) Box plot showing activation in the left vmPFC for the Unpleasant > Neutral contrast across the misophonia (MISO), hyperacusis (HY), comorbid (MiHy), and control (CTR) groups. Omnibus tests indicated marginal significance. Exploratory post hoc comparisons revealed greater activation in MISO compared with CTR. Box plot displays corresponding contrast estimates across groups. *p < 0.05;** p< 0.01; ***p < 0.001.

In the network-wise ROI analysis, group differences emerged in the default mode and central executive network ROIs. In the default mode network, a marginally significant Group x Condition (2 levels: U>N and P>N) interaction was observed (*Pillai’s Trace* = 0.349; *F*(9,261) = 1.766; *p* = 0.074). Exploratory post hoc tests indicated greater activation in the left vmPFC in MISO compared with CTR for U>N contrast (*T* = 2.01; adjusted *p* = 0.048; Fig. 3), although this effect should be interpreted with caution given the non-significant omnibus test. Furthermore, the HY and MiHy groups did show activation differences in vmPFC compared with other groups. These results are summarized in Table 3. In the central executive network, a significant Group × Condition interaction reached significance (*Pillai’s Trace* = 0.182, *F*(6, 174) = 2.90, *p* = 0.01). However, univariate follow-ups for left and right dorsolateral prefrontal cortex did not reveal significant effects.

### Functional connectivity analyses

Seed-to-voxel functional connectivity analyses for the U>N contrast revealed significant group differences within the salience, central executive, and emotion networks.

#### Salience network

A multivariate F-test across four salience network seeds (left and right anterior insula [AI], left and right anterior cingulate cortex [ACC]) revealed a significant group effect on connectivity between the salience network and a cluster encompassing the superior division of the left lateral occipital cortex (sLOC) and the left angular gyrus (AG) (*F*(12, 223) = 2.89, p_FWE_ = 0.0032). Follow-up univariate F-tests showed significant group effects for the left AI and bilateral ACC seeds.

**Fig. 4:**
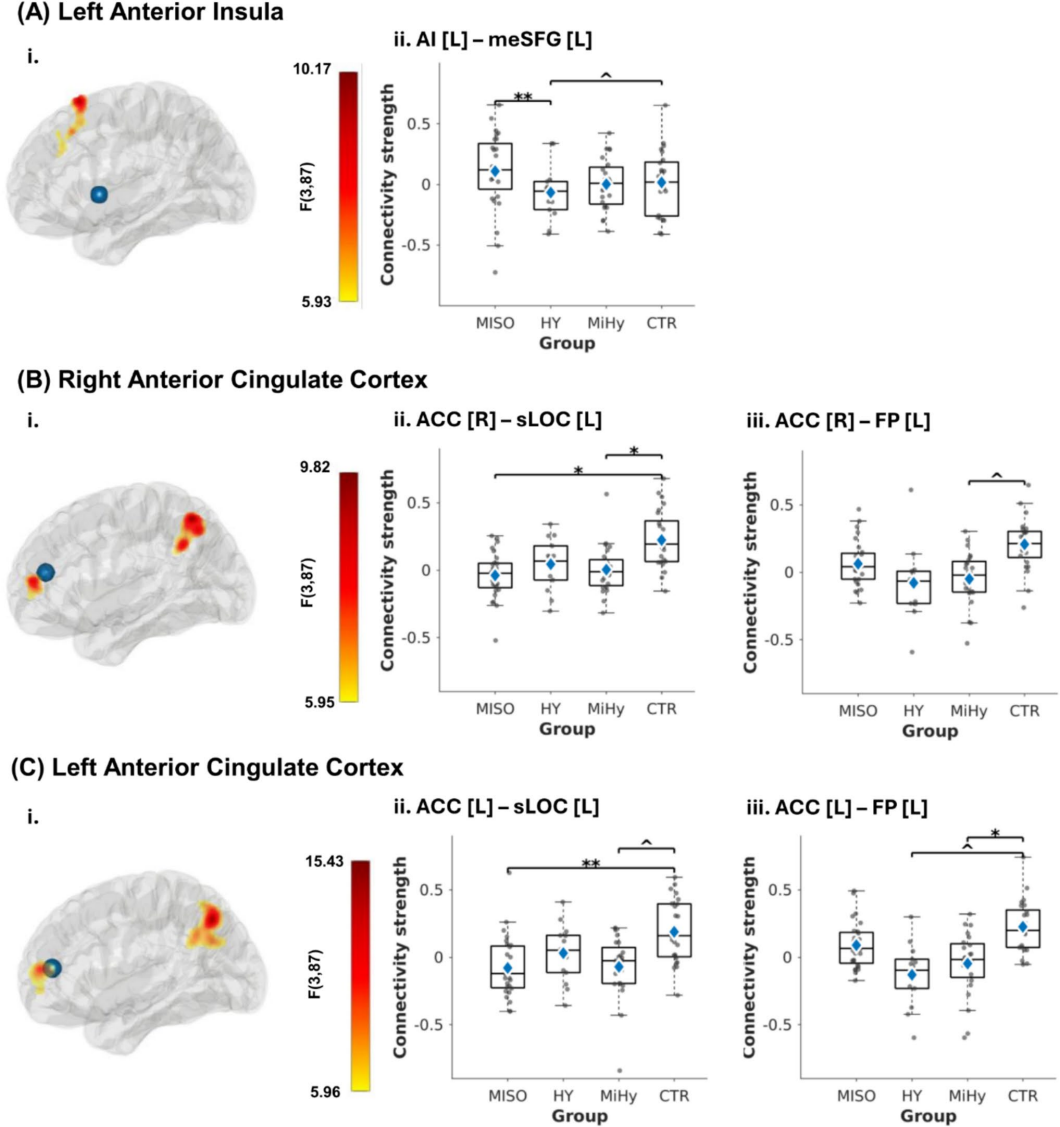
Seed-based functional connectivity of salience network regions across misophonia (MISO), hyperacusis (HY), comorbid (MiHy), and control (CTR) groups for the Unpleasant > Neutral contrast. Salience network included bilateral anterior insula (AI) and anterior cingulate cortex (ACC) seeds, with significant group differences observed for the left AI and bilateral ACC seeds. **A–C** Results for the left AI, right ACC, and left ACC seeds, respectively. For each seed, statistical maps are displayed on a glass brain (**Ai**, **Bi**, **Ci**; hot colors indicate F-values; blue sphere indicates the seed) and corresponding box plots show connectivity values between the seed and target regions across groups (**Aii**: left AI–left medial superior frontal gyrus [meSFG]; **Bii**: right ACC–left superior lateral occipital cortex [sLOC]; **Biii**: right ACC–left frontal pole [FP]; **Cii**: left ACC–left sLOC; **Ciii**: left ACC–left FP). R and L on the box plots indicate right and left hemispheres, respectively. All results are reported at cluster-level FWE-corrected *p *< 0.05 and Bonferroni-corrected for multiple comparisons. Blue diamonds indicate group means. *p < 0.05; **p < 0.01; ***p < 0.001; ^p denotes pairwise comparisons significant before Bonferroni correction

Connectivity between the left AI seed and the left medial superior frontal gyrus (meSFG) varied significantly across groups (*F*(3,87) > 5.93, p_FWE_ = 0.026) (Fig. 4Ai). Figure 4Aii displays boxplot of the beta values at the global maximum in the left meSFG (MNI coordinates: −8, 20, 66) for each group. The HY group exhibited the lowest average connectivity. Post-hoc comparisons revealed reduced AI-meSFG connectivity in HY relative to MISO (p_FWE_ = 0.0012). The reduction relative to CTR (p_FWE_ = 0.030) did not survive Bonferroni correction. Furthermore, the MiHy group did not exhibit significant differences in AI–meSFG connectivity relative to any other group.

The connectivity patterns of both ACC seeds showed similar group effects. For the right ACC seed, group differences were observed in connectivity with two clusters: left sLOC extending into the AG (*F*(3,87) > 5.93, p_FWE_ = 0.0091) and, at a trend level, the left frontal pole/anterior prefrontal cortex (*F*(3,87) > 5.93, p_FWE_ = 0.030) (Fig. 4Bi). Figure 4Bii and Biii show boxplots for beta values at the global maxima in left sLOC/AG (MNI coordinates: −46, −60, 56) and left frontal pole (MNI coordinates: −40, 54, 10), respectively. Post-hoc tests indicated reduced right ACC–left sLOC/AG connectivity in MISO (p_FWE_ = 0.0080) and MiHy (p_FWE_ = 0.0064) groups compared with CTR. No significant differences in right ACC–left sLOC/AG connectivity were observed for the HY group relative to any other group. Connectivity between the right ACC and left frontal pole was lower only in the MiHy group compared to CTR (p_FWE_ = 0.0372), but this effect did not survive Bonferroni correction and is therefore reported as a trend.

Similarly, the left ACC seed showed significant group differences in connectivity with two clusters: the left sLOC/AG (*F*(3,87) > 5.93, p_FWE_ = 0.0016) and, at a trend level, the left frontal pole (*F*(3,87) > 5.93, p_FWE_ = 0.0152) (Fig. 4Ci). Figure 4Cii and Ciii display the boxplots of the beta values at global maxima in left sLOC/AG (MNI coordinates: −44, −66, 46) and left frontal pole (MNI coordinates: −40, 56, 10), respectively. Post-hoc tests revealed reduced left ACC–sLOC/AG connectivity in MISO compared with CTR (p_FWE_ = 0.0012) and a trend-level reduction in MiHy relative to CTR (p_FWE_ = 0.048). The HY group did not reveal any differences in left ACC–sLOC/AG connectivity compared with other groups. The left frontal pole cluster exhibited significantly reduced connectivity with the left ACC in MiHy compared with CTR (p_FWE_ = 0.0078), and HY showed a trend-level reduction (p_FWE_ = 0.036) that did not survive Bonferroni correction. The MISO did not exhibit differences in left ACC–left frontal pole connectivity compared with other groups. Summary of group comparisons across salience network seeds are provided in Table 1.

**Table:** Seed-to-voxel functional connectivity results for seeds in Salience and Central Executive network during the Unpleasant > Neutral contrast, showing significant connectivity differences among four groups (misophonia only [MISO]; hyperacusis only [HY]; comorbid misophonia and hyperacusis [MiHy]; controls [CTR])

Network	Seed	Group comparison	Region	Hemisphere	MNI			Cluster size (k)	*T*
					**x**	**y**	**z**		
Salience	Left AI	HY<MISO	Medial Superior Frontal Gyrus	Left	−8	20	66	1180	5.52
		HY<CTR	Medial Superior Frontal Gyrus	Left	−6	26	44	329	3.41*
	Left ACC	MISO < CTR	Superior Lateral Occipital Cortex	Left	−44	−66	46	1327	5.4
		HY < CTR	Frontal Pole	Left	−40	56	12	295	3.41*
		MiHy < CTR	Superior Lateral Occipital Cortex	Left	−44	−66	48	243	3.41*
			Frontal Pole	Left	−34	58	8	581	4.67
	Right ACC	MISO < CTR	Superior Lateral Occipital Cortex	Left	−46	−60	56	606	4.71
		MiHy < CTR	Superior Lateral Occipital Cortex	Left	−42	−58	52	653	4.71
			Frontal Pole	Left	−40	52	10	296	3.41*
Central executive	Left dlPFC	MISO > CTR	Supplementary Motor Cortex	Left	−2	2	54	798	5.1
		HY > CTR	Supplementary Motor Cortex	Left	−6	−6	54	241	4.35
		HY < CTR	Cerebellar Crus I	Left	−40	−68	−42	346	4.15*
		HY <MISO	Cerebellar Crus I	Left	−34	−60	−32	854	5.9

AI = anterior insula; ACC anterior cingulate cortex; dlPFC dorsolateral prefrontal cortex

^*^Results significant before pairwise Bonferroni correction for multiple *t*-tests

#### Central executive network

**Fig. 5:**
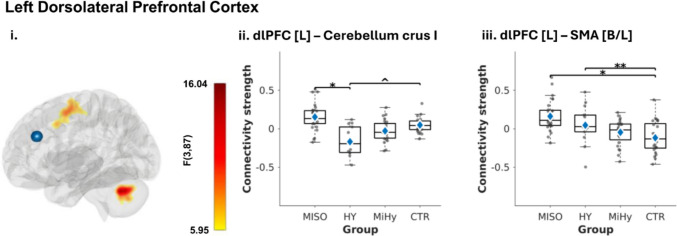
Seed-based functional connectivity of central executive network regions across misophonia (MISO), hyperacusis (HY), comorbid (MiHy), and control (CTR) groups for the Unpleasant > Neutral contrast. Central executive network included bilateral dorsolateral prefrontal cortex (dlPFC) seeds, with significant group differences observed for left dlPFC. (**i**) Statistical map on a glass brain (hot colors indicate F-values; blue sphere indicates the seed). (**ii** and **iii**) Box plots of connectivity beta values for left dlPFC–left cerebellar Crus I, and left dlPFC–bilateral supplementary motor area (SMA) connectivity, respectively. B/L and L on the box plots indicate bilateral and left hemispheres, respectively. All results are reported at cluster-level FWE-corrected *p *< 0.05 and Bonferroni-corrected for multiple comparisons. Blue diamonds indicate group means. *p < 0.05; **p < 0.01; ***p < 0.001; ^p denotes pairwise comparisons significant before Bonferroni correction

A multivariate F-test using bilateral dorsolateral prefrontal cortex (dlPFC) seeds revealed a significant group effect on connectivity with the left cerebellum crus I (*F*(6,172) > 3.95, p_FWE_ = 0.024) and bilateral supplementary motor area (SMA; *F*(6,172) > 3.95, p_FWE_ = 0.049). Follow-up univariate analyses indicated that the group effects were driven by the left dlPFC. Specifically, significant group differences were observed for left dlPFC connectivity with two clusters: the left cerebellum crus I (*F*(3,87) > 5.93, p_FWE_ = 0.0044) and the bilateral SMA (*F*(3,87) > 5.93, p_FWE_ = 0.0070) (Fig. 5i). Figure 5ii shows the boxplot of beta values at the global maximum in cerebellum crus I (MNI coordinates: −32, −60, −32), where the HY group exhibited the lowest average connectivity. Bonferroni-corrected post hoc comparisons confirmed significantly reduced left dlPFC–cerebellum crus I connectivity in HY relative to MISO (p_FWE_ = 0.0052). A trend-level reduction was also observed in HY relative to CTR (p_FWE_ = 0.017). Left dlPFC–cerebellum Crus I connectivity did not significantly differ in the MiHy group compared with any other group. Figure 5iii shows box plots of beta values at the global maximum in SMA (MNI: −2, 2, 54), where the CTR group exhibited the lowest average connectivity. Both MISO (*p* = 0.0058) and HY (*p* = 0.0006) showed significantly greater dlPFC–SMA connectivity relative to CTR. In the HY group, this hyperconnectivity further extended into the bilateral precentral and postcentral gyri. No significant differences were observed between the MiHy and CTR groups. Summary of group comparisons across central executive network seeds are provided in Table 1.

#### Emotion processing network

A multivariate F-test for bilateral amygdalae seeds indicated group differences in connectivity with the left intracalcarine cortex (*F*(6,172) > 3.95, p_FWE_ = 0.0064), with the global maximum located at MNI coordinates −8, 78, 12. However, follow-up univariate analyses did not reveal significant group effects.

### Exploratory correlation analysis

**Fig. 6:**
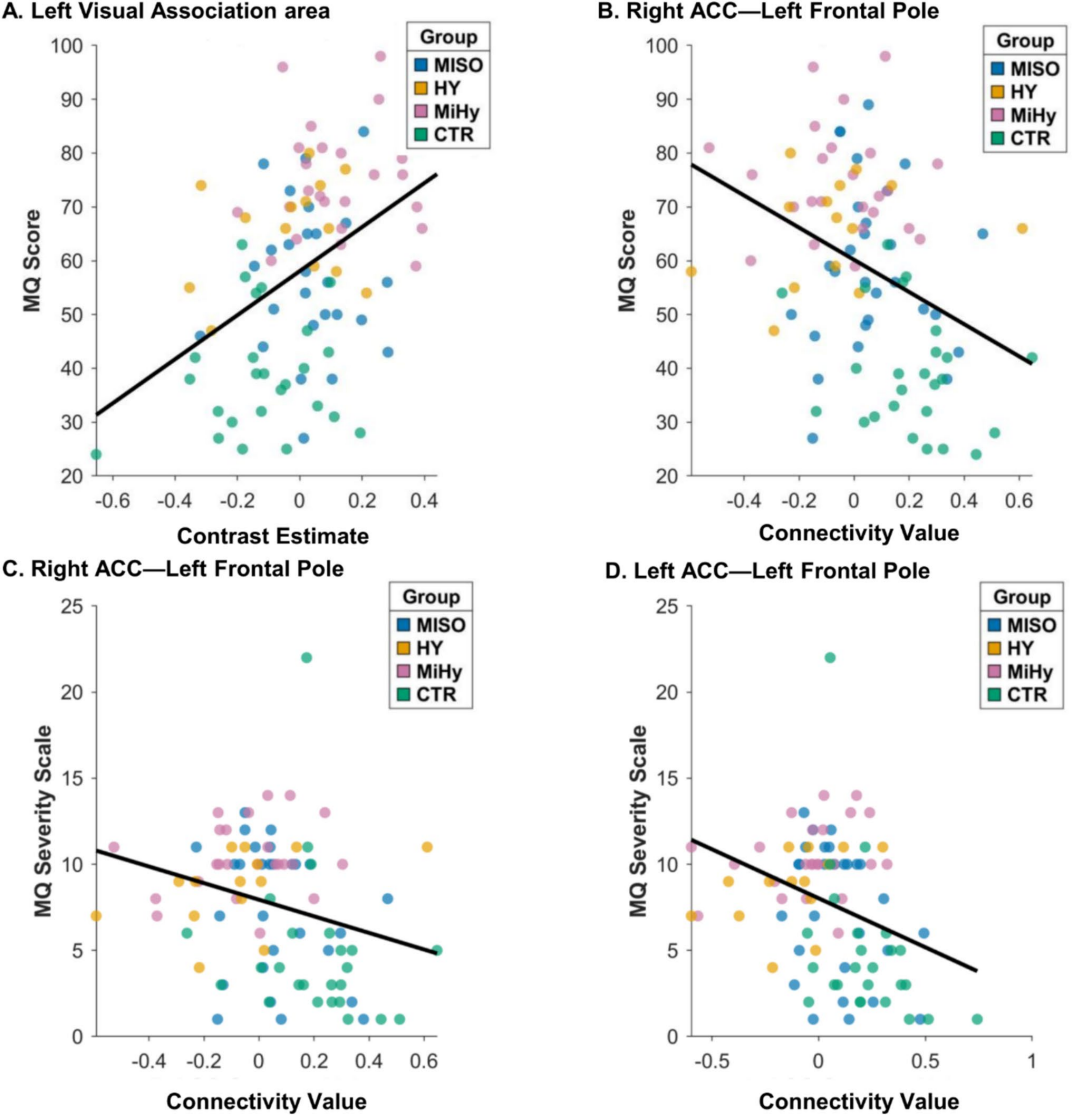
Correlations between misophonia questionnaire scores and brain measures. (**A**) Misophonia questionnaire total scores and contrast estimates in the left visual association area (peak MNI: −20, −90, 28) from whole-brain functional activation analysis. (**B**) Misophonia questionnaire total scores and seed-to-voxel functional connectivity between the right anterior cingulate cortex (ACC) seed and left frontal pole (FP) (peak MNI: −40, 52, 10). (**C**) Misophonia questionnaire severity scale and seed-to-voxel connectivity between right ACC seed and left FP voxel (peak MNI: −40, 52, 10). (**D**) Misophonia questionnaire severity scale and seed-to-voxel connectivity between left ACC seed and left FP voxel (peak MNI: −34, 58, 8)

We examined correlations between misophonia symptom severity (MQ total and severity scores) and brain activation and connectivity measures. Contrast estimates for the U > N condition were extracted from significant clusters in the secondary visual and visual association cortices identified in the whole-brain analysis. While no significant correlations were found within individual groups, pooling all participants revealed a significant positive correlation (Spearman’s ρ = 0.315, adjusted *p* = 0.004), indicating that greater misophonia severity is associated with increased activation in higher-order visual areas (Fig. 6).

We also examined correlations between misophonia symptom severity and connectivity values from key seed-to-voxel pairs discussed above: left AI–meSFG, left and right ACC–sLOC/AG, left and right ACC–left frontal pole, left dlPFC–bilateral SMA, and left dlPFC–cerebellar Crus I. No significant correlations emerged within individual groups. However, when all groups were pooled together, we observed a significant negative correlation between MQ total scores and right ACC–frontal pole connectivity (ρ = –0.385, adjusted *p* = 0.0023). Similarly, MQ severity scores negatively correlated with connectivity between both right ACC–frontal pole (ρ = –0.301, adjusted *p* = 0.05) and left ACC–frontal pole (ρ = –0.338, adjusted *p* = 0.049) (Fig. 6). Given the fundamental differences in MQ scores across groups, these correlations should be interpreted cautiously, as the findings may partly reflect group differences rather than purely continuous relationships.

## Discussion

The present study investigated differences in brain activation and connectivity between misophonia and hyperacusis, two distinct but often comorbid sound sensitivity disorders. Prior research has rarely compared the two conditions directly, has often overlooked comorbidity, and has relied on heterogeneous stimuli, making it difficult to distinguish shared versus disorder-specific mechanisms. To address these gaps, we employed task-based fMRI paradigm using standardized affective sounds. We examined neural responses to self-rated unpleasant, pleasant, and neutral sounds in four well-defined groups: misophonia only (MISO), hyperacusis only (HY), comorbid misophonia and hyperacusis (MiHy), and healthy controls (CTR). Our findings revealed both distinct and overlapping patterns across groups: 1) MISO and MiHy showed increased activation in visual association area and reduced visual–salience network connectivity; 2) HY and MiHy exhibited reduced salience–prefrontal network connectivity; 3) the MiHy group exhibited a pattern similar to both MISO (increased visual activation and reduced visual–salience connectivity) and HY (reduced salience–prefrontal connectivity) groups; and 4) both MISO and HY showed increased prefrontal–motor connectivity. These findings are discussed next.

### Visual cortex hyperactivity and diminished functional connectivity with ACC in misophonia

In our study, the MiHy group, relative to CTR, showed robust hyperactivation of the left visual association area, extending bilaterally into secondary visual cortices for unpleasant versus neutral sounds. A similar but weaker effect was also observed in the MISO group. Furthermore, both misophonia groups (MISO and MiHy), relative to CTR, exhibited reduced functional connectivity between the bilateral ACC and left sLOC (visual association area) for self-rated unpleasant versus neutral sounds. These patterns were not observed in the HY group, suggesting that visual cortex hyperactivity and reduced functional connectivity between the ACC and visual areas are neural patterns more strongly associated with misophonia.

Evidence from both task-based and resting-state functional connectivity studies have implicated atypical visual cortex involvement in misophonia. Schröder et al. (2019) reported reduced fusiform gyrus activation—a region critical for face perception (Kanwisher & Yovel, 2006) in a symptom provocation paradigm. Although differences in stimulus modality across conditions (visual for neutral clips versus audiovisual for trigger and unpleasant sounds) complicate interpretation, their findings nonetheless indicate atypical visual cortex engagement in misophonia. Complementing this, resting-state functional connectivity evidence from Eijsker et al. (2021) revealed stronger connectivity within an independent component encompassing lateral occipital cortex, part of the ventral attention network. More recently, Ajmera et al. (2025), using a large-scale, machine-learning–driven resting-state analysis on the same groups of participants as in the present study, reported that disruptions in networks supporting higher-order visual processing were observed in comorbid misophonia–hyperacusis groups. Our results extend these observations by showing increased activation in visual association areas and reduced ACC–visual functional connectivity during generally unpleasant (not triggers) sound processing.

Emotionally salient sounds have been shown to reflexively engage higher-order visual cortex even in the absence of concurrent visual input (McDonald et al., 2013), reflecting typical cross-modal sensory interactions that may facilitate emotional sound processing. The ACC is a key node in detecting salient stimuli and exerting top-down modulatory influences over sensory cortices, including visual areas (Crottaz-Herbette & Menon, 2006). In individuals with misophonia, regardless of comorbid hyperacusis, we observed reduced functional connectivity between the ACC and visual association areas during processing of unpleasant sounds. This reduced connectivity may reflect altered coordination between salience detection and visual processing systems, potentially contributing to the heightened visual cortex activation observed in misophonia.

Altered connectivity between the ACC and visual association areas during emotional sound processing may underlie how visual context influences auditory emotional responses. Behavioral evidence shows that pairing misophonic trigger sounds with positive or incongruent visual stimuli reduces unpleasantness, bodily sensations, and emotional intensity more strongly in individuals with misophonia than controls (Heller et al., 2025; Mahzouni et al., 2024; Samermit et al., 2022; Siepsiak et al., 2023). These findings highlight the modulatory role of visual context in auditory affective processing. This cross-modal interaction suggests potential clinical avenues for interventions leveraging visual cues to alleviate maladaptive emotional responses in misophonia.

Together, these findings suggest that altered cross-modal activity in visual association areas may represent a stable trait of misophonia (independent of comorbid hyperacusis), evident across both resting-state (Eijsker et al., 2021) and task-based contexts. Importantly, this atypical visual processing is not restricted to misophonia-specific trigger sounds but also occurs in response to other unpleasant auditory stimuli. However, further research is necessary to better understand the extent, underlying mechanisms, and functional implications of these alterations across diverse misophonia populations, varying in severity, age, and comorbidities.

### Reduced connectivity in salience and executive control networks in hyperacusis

Previous neuroimaging research on hyperacusis has predominantly focused on ascending auditory pathways, emphasizing increased central gain as a core mechanism (Auerbach et al., 2014; Gu et al., 2010; Koops & van Dijk, 2021). However, this auditory-centric perspective overlooks the involvement of extra-auditory networks that contribute to the disorder’s broader sensory and affective symptoms. Supporting this broader view, Chen et al. (2015) reported increased connectivity within auditory-limbic-arousal-cerebellar network in a mouse model of hyperacusis. Similarly, Smith et al. (2025) identified autonomic emotional signatures, such as involuntary pupil dilation and facial movements, that correlate with sound sensitivity severity in humans, implicating affective and regulatory brain systems beyond primary auditory pathways.

Consistent with evidence implicating extra-auditory networks in hyperacusis, the HY group in our study showed reduced functional connectivity between the left AI and meSFG (relative to CTR and MISO). Connectivity between the left ACC and frontal pole was also lower in HY compared to CTR during self-rated unpleasant versus neutral sound processing. These disruptions suggest reduced integration between salience detection and higher-order cognitive regions involved in executive control, social cognition, and self-reflective processing (Goldberg et al., 2006; Levy, 2024). HY group also showed reduced left dlPFC and cerebellar Crus I functional connectivity, suggesting disruptions in cerebro-cerebellar circuits that support complex cognitive and affective regulation (Stoodley & Schmahmann, 2010; van Overwalle et al., 2014). Together, these findings suggest that hyperacusis involves disrupted coordination between salience and cognitive control networks during affective evaluation of emotionally aversive sounds.

Connectivity of salience and executive control regions was largely preserved in the MISO group, showing patterns comparable to controls (Figs. 4Aii and 5ii). Because the task used general unpleasant rather than individualized trigger sounds, this preservation may indicate that higher-order control mechanisms remain intact during nontrigger auditory processing in misophonia. By contrast, hyperacusis may involve disrupted top-down modulation during emotional appraisal regardless of sound type, consistent with its broader auditory hypersensitivity. This distinction highlights differential patterns of functional connectivity across the two conditions and that could inform tailored intervention strategies and serve as neural biomarkers for tracking treatment response.

### Neural patterns in the comorbid misophonia and hyperacusis group

The MiHy group exhibited activation and connectivity profiles that overlapped with those observed in both MISO and HY during self-rated unpleasant versus neutral sound processing. Specifically, MiHy showed robust hyperactivation in visual association cortices relative to CTR, resembling patterns observed in MISO. MiHy also exhibited reduced ACC–visual association area connectivity (relative to CTR), paralleling MISO. In addition, MiHy showed reduced ACC–frontal pole connectivity (relative to CTR), similar to the HY group but more pronounced. These results indicate concurrent alterations in visual processing, salience–visual coupling, and salience–cognitive control interactions in MiHy group, consistent with neural patterns associated with both misophonia and hyperacusis. This aligns with behavioral evidence from Andermane et al. (2023), who suggest that individuals with comorbid misophonia and hyperacusis exhibit patterns where hyperacusis-related emotional appraisal are superimposed as an additional layer on misophonia. Future work should determine whether MiHy represents a straightforward overlap, a more complex interaction of misophonia and hyperacusis, or a distinct phenotype. Such studies would also benefit from incorporating individualized misophonic trigger sounds, particularly in misophonia and comorbid groups, to better understand interactions between the conditions. Clinically, this comorbidity may require interventions addressing both selective and generalized auditory sensitivities.

### Sensorimotor network engagement in both misophonia and hyperacusis

Our findings revealed that both MISO and HY exhibited increased left dlPFC—bilateral SMA connectivity relative to CTR during self-rated unpleasant versus neutral sound processing, suggesting shared engagement of motor-related areas across both disorders. This interpretation is supported by converging evidence from both disorders: structural and functional imaging in hyperacusis shows reduced gray matter and cortical thickness in the SMA alongside heightened sound-evoked responses (Makani et al., 2023), while misophonia research demonstrates sensory-motor involvement during task-based and resting-state paradigms (Hansen et al., 2022; Kumar et al., 2021). Furthermore, dlPFC–SMA coupling reflects enhanced integration between executive control and motor planning systems (Nachev et al., 2008), indexing a state of motor readiness. This dlPFC–SMA coupling may underlie fight-or-flight response to aversive sounds, representing a shared mechanism in both disorders. Notably, this increased dlPFC–SMA connectivity was not observed in the MiHy group for reasons that remain unclear and require further investigation.

### Associations between brain activation, functional connectivity, and misophonia severity

No significant associations between symptom severity and brain activation or functional connectivity were found within individual groups. However, pooling participants from all the groups revealed significant relationships (note that the controls scored low on the misophonia scales). Specifically, greater misophonia severity correlated with increased activation in higher-order visual cortices during unpleasant versus neutral sound processing. This extends our group-level findings of heightened visual association area activation in the MISO and MiHy groups and suggests that symptom severity may be associated with the degree of cross-modal visual engagement during affective auditory processing. Additionally, higher misophonia severity was linked to reduced functional connectivity between the bilateral anterior cingulate cortex and left frontal pole, regions involved in cognitive control during affective evaluation. This pattern may indicate diminished top-down regulatory capacity as symptoms worsen, potentially contributing to challenges in modulating emotional responses to aversive sounds.

While these correlations offer preliminary insights into brain-behavior relationships, they should be interpreted with caution. Activation and connectivity values were derived from clusters identified in group-level analyses, raising concerns about non-independence. Thus, these findings are exploratory and future studies using independent datasets or continuous symptom-based designs are needed to confirm their robustness and specificity.

### Limitations

Several limitations should be acknowledged. The sample size for this study was guided by prior task-based fMRI research in misophonia (Kumar et al., 2017, 2021; Schröder et al., 2019); however, an explicit a priori power analysis was not conducted. Although our total sample size (N = 91) allowed analyses across four groups (MISO, HY, MiHy, and CTR), the relatively small hyperacusis group (n = 14) may have reduced statistical power to detect effects specific to this subgroup. Additionally, restricting the sample to young adults aged 18 to 25 helped control for age but limits generalizability across the lifespan and may not capture the full clinical spectrum of misophonia and hyperacusis. Furthermore, the binary classification of misophonia and the use of HQ scores for group assignment limited our ability to examine symptom severity as a continuous dimension. Interestingly, the HY group showed elevated MQ scores compared with the MISO group despite not meeting clinical criteria for misophonia based on structured interview. This suggests that the MQ may lack specificity, reflecting broader sound intolerance rather than misophonia alone. Supporting this, recent psychometric evaluations indicate that several measurement properties of the MQ remain insufficiently established (Kula et al., 2022; Maleki et al., 2025). Although exploratory correlations between neural measures and MQ scores were conducted, significant associations were not observed within individual diagnostic groups and emerged only when analyses were pooled across the full sample, raising concerns that these effects may be driven by between-group differences. These findings should therefore be interpreted cautiously and highlight the need for more sensitive, continuous assessment tools in future research.

For the task, we selected standardized affective sounds from the IADS-2 database and excluded commonly reported misophonic trigger sounds (e.g., human-generated orofacial sounds). However, because misophonic triggers are individualized and subjective trigger appraisals were not collected during the task, it remains possible that some stimuli may be experienced as trigger-like by a subset of participants. In addition, the relatively short inter-stimulus interval (1.5 s) may have allowed affective responses, particularly to unpleasant sounds, to persist across trials. Although this timing is comparable to a prior task-based fMRI paradigm (Schröder et al., 2019; 2 s interstimulus interval), residual affect may have influenced subsequent evaluations and contributed to the relatively low number of pleasant-rated trials across groups. While carryover effects were likely similar across groups and thus less likely to drive group differences, the current design was not intended to isolate these effects. Future work using longer inter-stimulus intervals could directly examine carryover dynamics in sound tolerance disorders. Relatedly, the limited number of pleasant-rated trials constrained analyses of positive affective processing primarily to ROI-level effects. Finally, the use of continuous fMRI acquisition introduced scanner noise, which could have influenced participants’ emotion perception, despite sounds being presented at a loud but comfortable level. Importantly, several prior neuroimaging studies on misophonia and hyperacusis have employed similar continuous acquisition methods and have reported robust and consistent findings (Hofmeier et al., 2021; Kumar et al., 2017, 2021; Schröder et al., 2019), supporting the validity of this approach. Future studies might employ sparse-sampling fMRI acquisition methods to minimize auditory interference and improve sensitivity; however, this strategy can introduce activations related to onset and offset of scanner noise and reduces statistical power due to fewer images being acquired.

## Conclusions

Our study elucidates both overlapping and distinct neural correlates associated with misophonia, hyperacusis, and their co-occurrence during affective processing of self-rated unpleasant sounds. Misophonia was primarily associated with atypical cross-modal visual activation and altered visual–salience network connectivity, regardless of comorbid hyperacusis. In contrast, hyperacusis showed reduced functional integration between salience and executive control networks, suggesting potential disruptions in top-down modulation during emotional appraisal of generally unpleasant sounds. This connectivity appeared largely preserved in misophonia, which may reflect relatively intact top-down control for generally unpleasant (not trigger) sounds. These differences are consistent with the more selective sound reactivity characteristic of misophonia versus the broader auditory sensitivity typical of hyperacusis. The comorbid group exhibited neural features of both disorders. Both hyperacusis and misophonia exhibited increased dlPFC–SMA connectivity, suggesting a possible shared mechanism of heightened motor readiness that may underlie avoidance or defensive responses. Collectively, these findings reveal overlapping and disorder-specific neural patterns across sound tolerance profiles, potentially informing more precise diagnosis and targeted interventions for sound intolerance disorders. However, as these results reflect group-level differences, future research should investigate individual variability and symptom dimensions to better elucidate underlying mechanisms.

## Supplementary Information

Below is the link to the electronic supplementary material.

Supplementary file1 (DOCX 30 KB)

## Data Availability

The data supporting the findings of this study will be made available to researchers upon reasonable request to the corresponding author after the publication of the manuscript.

## Code availability

No custom code or software was used in this study.
