# Supplementary material for: Differential brain responses to affective sounds in misophonia and hyperacusis: A task-based fMRI approach
Source: Cogn Affect Behav Neurosci. 2026 Apr 14;26(4):1741–60. doi: 10.3758/s13415-026-01435-z (PMC13385084; doi:10.3758/s13415-026-01435-z)
Supplement: Supplementary file 1 — Supplementary file1 (DOCX 30 KB) [file 13415_2026_1435_MOESM1_ESM.docx]

**Supplementary**

**Table S1.** List of International Affective Digitized Sounds-2 (IADS-2) sound stimuli included in the study and their normative valence and arousal scores (mean ± SD).

| **Sl.**  **No** | **Sound ID** | **Description** | **Valence Category** | **Pleasure (mean)** | **Pleasure (SD)** | **Arousal (mean)** | **Arousal (SD)** |
| --- | --- | --- | --- | --- | --- | --- | --- |
|  | 105 | Puppy | Unpleasant | 2.88 | 2.14 | 6.4 | 2.14 |
|  | 106 | Growl1 | Unpleasant | 3.37 | 1.64 | 6.39 | 1.64 |
|  | 115 | Bees | Unpleasant | 2.16 | 1.33 | 7.03 | 1.33 |
|  | 116 | Buzzing | Unpleasant | 3.02 | 1.65 | 6.51 | 1.65 |
|  | 134 | Rattle Snake | Unpleasant | 3.55 | 1.99 | 6.98 | 1.99 |
|  | 255 | Vomit | Unpleasant | 2.08 | 1.78 | 6.59 | 1.78 |
|  | 260 | BabiesCry | Unpleasant | 2.04 | 1.39 | 6.87 | 1.39 |
|  | 261 | Baby Cry | Unpleasant | 2.75 | 1.68 | 6.51 | 1.68 |
|  | 278 | Child Abuse | Unpleasant | 1.57 | 1.43 | 7.27 | 1.43 |
|  | 281 | Attack3 | Unpleasant | 3.43 | 2.63 | 7.33 | 2.63 |
|  | 282 | Fight2 | Unpleasant | 2.92 | 2.34 | 7.2 | 2.34 |
|  | 284 | Attack3 | Unpleasant | 2.01 | 1.48 | 7.05 | 1.48 |
|  | 288 | Creep | Unpleasant | 2.71 | 1.75 | 6.82 | 1.75 |
|  | 289 | Gun Shot | Unpleasant | 3.08 | 1.71 | 6.57 | 1.71 |
|  | 292 | Male Scream | Unpleasant | 1.99 | 1.41 | 7.28 | 1.41 |
|  | 310 | Crowd1 | Unpleasant | 3.89 | 2.32 | 6.78 | 2.32 |
|  | 312 | Crowd3 | Unpleasant | 3.89 | 2.13 | 6.89 | 2.13 |
|  | 420 | Car Horns | Unpleasant | 2.34 | 1.51 | 7.08 | 1.51 |
|  | 422 | Tire Skids | Unpleasant | 2.22 | 1.47 | 7.52 | 1.47 |
|  | 501 | Plane Crash | Unpleasant | 2.74 | 1.76 | 6.93 | 1.76 |
|  | 600 | Bike Wreck | Unpleasant | 2.13 | 1.55 | 7.28 | 1.55 |
|  | 624 | Air Raid | Unpleasant | 2.82 | 1.75 | 7.1 | 1.75 |
|  | 625 | May Day | Unpleasant | 3.35 | 2.03 | 6.94 | 2.03 |
|  | 626 | Explosion | Unpleasant | 3.37 | 1.98 | 6.61 | 1.98 |
|  | 709 | Alarm Clock | Unpleasant | 2.78 | 1.93 | 7.54 | 1.93 |
|  | 711 | Siren1 | Unpleasant | 2.61 | 1.59 | 7.39 | 1.59 |
|  | 713 | Sirens | Unpleasant | 2.95 | 1.71 | 6.98 | 1.71 |
|  | 714 | Siren2 | Unpleasant | 3.1 | 1.67 | 6.94 | 1.67 |
|  | 719 | Dentist Drill | Unpleasant | 2.89 | 1.67 | 6.91 | 1.67 |
|  | 732 | Crash | Unpleasant | 2.89 | 1.68 | 6.98 | 1.68 |
|  |  |  |  |  |  |  |  |
|  | 102 | Cat | Neutral | 4.63 | 2.17 | 4.91 | 2.17 |
|  | 104 | Panting | Neutral | 4.96 | 1.68 | 5.37 | 1.68 |
|  | 107 | Dog | Neutral | 5.47 | 2.22 | 5.85 | 2.22 |
|  | 113 | Cows | Neutral | 5.45 | 1.71 | 4.88 | 1.71 |
|  | 120 | Rooster | Neutral | 5.2 | 2.1 | 5.41 | 2.1 |
|  | 130 | Pig | Neutral | 4.64 | 2.11 | 4.93 | 2.11 |
|  | 132 | Chickens | Neutral | 5.64 | 1.76 | 4.77 | 1.76 |
|  | 170 | Night | Neutral | 5.31 | 2.12 | 4.6 | 2.12 |
|  | 171 | Country Night | Neutral | 5.59 | 1.79 | 3.71 | 1.79 |
|  | 245 | Hiccup | Neutral | 4.18 | 1.85 | 5.05 | 1.85 |
|  | 246 | Heartbeat | Neutral | 4.83 | 1.81 | 4.65 | 1.81 |
|  | 252 | Male Snore | Neutral | 4.01 | 1.87 | 4.75 | 1.87 |
|  | 320 | Office1 | Neutral | 4.23 | 1.56 | 5.48 | 1.56 |
|  | 322 | Typewriter | Neutral | 5.01 | 1.82 | 4.79 | 1.82 |
|  | 358 | Writing | Neutral | 4.52 | 1.34 | 4.87 | 1.34 |
|  | 376 | Lawnmower | Neutral | 4.88 | 1.62 | 4.6 | 1.62 |
|  | 382 | Shovel | Neutral | 4.33 | 1.42 | 4.64 | 1.42 |
|  | 410 | Helicopter2 | Neutral | 4.86 | 1.48 | 5.89 | 1.48 |
|  | 425 | Train | Neutral | 5.09 | 1.42 | 5.15 | 1.42 |
|  | 500 | Wind | Neutral | 4.32 | 2.03 | 5.4 | 2.03 |
|  | 627 | Rain1 | Neutral | 4.83 | 1.89 | 4.65 | 1.89 |
|  | 700 | Toilet | Neutral | 4.68 | 1.61 | 4.03 | 1.61 |
|  | 701 | Fan | Neutral | 4.95 | 1.47 | 4.41 | 1.47 |
|  | 708 | Clock | Neutral | 4.34 | 1.42 | 3.51 | 1.42 |
|  | 720 | Brush Teeth | Neutral | 4.86 | 1.8 | 4.18 | 1.8 |
|  | 722 | Walking | Neutral | 4.83 | 1.22 | 4.97 | 1.22 |
|  | 723 | Radio | Neutral | 4.52 | 1.47 | 4.42 | 1.47 |
|  | 728 | Paper1 | Neutral | 4.72 | 1.26 | 4.35 | 1.26 |
|  | 729 | Paper2 | Neutral | 4.3 | 1.69 | 5.79 | 1.69 |
|  |  |  |  |  |  |  |  |
|  | 109* | Carousel | Pleasant | 6.4 | 2.13 | 5.64 | 2.13 |
|  | 110 | Baby | Pleasant | 7.64 | 2.1 | 6.03 | 2.1 |
|  | 111 | Music Box | Pleasant | 6.01 | 2.19 | 5.65 | 2.19 |
|  | 200 | Erotic Couple | Pleasant | 6.31 | 1.93 | 7.1 | 1.93 |
|  | 201 | EroticFem1 | Pleasant | 6.7 | 2.22 | 7.31 | 2.22 |
|  | 202 | EroticFem2 | Pleasant | 6.81 | 2.08 | 7.13 | 2.08 |
|  | 205 | EroticFem3 | Pleasant | 6.47 | 1.98 | 6.46 | 1.98 |
|  | 215 | Erotic Couple2 | Pleasant | 6.47 | 2.12 | 7.32 | 2.12 |
|  | 220 | Boy Laugh | Pleasant | 7.28 | 1.91 | 6 | 1.91 |
|  | 224 | Kids2 | Pleasant | 6.11 | 1.9 | 5.64 | 1.9 |
|  | 254 | Video Game | Pleasant | 6.17 | 1.65 | 5.58 | 1.65 |
|  | 311 | Crowd2 | Pleasant | 7.65 | 1.58 | 7.12 | 1.58 |
|  | 352 | Sports Crowd | Pleasant | 7.17 | 1.97 | 7.07 | 1.97 |
|  | 353 | Baseball | Pleasant | 7.38 | 1.53 | 6.62 | 1.53 |
|  | 355 | Crowd4 | Pleasant | 6.77 | 1.84 | 6.32 | 1.84 |
|  | 360 | Roller Coaster | Pleasant | 6.94 | 2.25 | 7.54 | 2.25 |
|  | 363 | Horse Race | Pleasant | 6.1 | 1.88 | 6.32 | 1.88 |
|  | 365 | Party | Pleasant | 6.97 | 1.9 | 6.32 | 1.9 |
|  | 366 | Casino1 | Pleasant | 7.09 | 1.73 | 6.26 | 1.73 |
|  | 367 | Casino2 | Pleasant | 7.33 | 1.74 | 6.72 | 1.74 |
|  | 378 | Doorbell | Pleasant | 6.06 | 2.01 | 6.15 | 2.01 |
|  | 415 | Countdown | Pleasant | 6.46 | 1.67 | 6.55 | 1.67 |
|  | 601 | Colonial Music | Pleasant | 6.53 | 1.66 | 5.84 | 1.66 |
|  | 716 | SlotMachine1 | Pleasant | 7 | 2.17 | 6.44 | 2.17 |
|  | 717 | SlotMachine2 | Pleasant | 7.32 | 1.64 | 6.56 | 1.64 |
|  | 808 | Bugle | Pleasant | 6.32 | 1.76 | 6.35 | 1.76 |
|  | 813 | Wedding | Pleasant | 7.2 | 1.86 | 5.89 | 1.86 |
|  | 815 | Rock N Roll | Pleasant | 7.9 | 1.53 | 6.85 | 1.53 |
|  | 817 | Bongos | Pleasant | 7.67 | 1.46 | 7.15 | 1.46 |
|  | 820 | Funk Music | Pleasant | 6.94 | 1.98 | 5.87 | 1.98 |

*Repeated twice
